# Readmission Trends Related to Unruptured Intracranial Aneurysm Treatment

**DOI:** 10.3389/fneur.2021.590751

**Published:** 2021-05-20

**Authors:** Tapan Mehta, Ninad Desai, Smit Patel, Shailesh Male, Adam Khan, Andrew Walker Grande, Ramachandra Prasad Tummala, Bharathi Dasan Jagadeesan

**Affiliations:** ^1^Department of Neurology and Interventional Neuroradiology, Hartford Hospital, Hartford, CT, United States; ^2^Department of Neurology, NYU Langone Medical Center, New York, NY, United States; ^3^Department of Neurology, University of Connecticut, Farmington, CT, United States; ^4^Department of Neurosurgery, Radiology and Neurology, University of Minnesota, Minneapolis, MN, United States

**Keywords:** unruptured, intracrainal, aneurysm, endovascular, clipping

## Abstract

**Background and Purpose:** Aneurysmal subarachnoid hemorrhage (SAH) is associated with high mortality. Prophylactic treatment of the unruptured intracranial aneurysm (UIA) is considered in a select group of patients thought to be at high for aneurysmal rupture. Hospital readmission rates can serve as a surrogate marker for the safety and cost-effectiveness of treatment options for UIAs; we present an analysis of the 30-day rehospitalization rates and predictors of readmission following UIA treatment with surgical and endovascular approaches.

**Methods:** We retrospectively analyzed data from the National Readmission Database (NRD) derived from the Healthcare Cost and Utilization Project for the year 2014. The cohort included patients with a primary discharge diagnosis of a treated unruptured aneurysm. The primary outcome variable was the 30-day readmission rate in open surgical vs. endovascularly treated groups. The secondary outcomes included predictors of readmissions, and causes of 30-day readmissions in these two groups.

**Results:** The 30-day readmission rate for the surgical group was 8.37% compared to 4.87% for the endovascular group. The index hospitalization duration was longer in the surgical group. A larger proportion of the patients readmitted following surgical treatment were hypertensive (76.35, vs. 63.43%), but the prevalence of other medical comorbidities was comparable in the two treatment groups.

**Conclusions:** There is a higher likelihood for 30-day readmission, longer duration of initial hospitalization and a lower likelihood of discharge home following surgical treatment of UIAs when compared to endovascular treatment. These findings, however, do not demonstrate long-term superiority of one specific treatment modality.

## Introduction

The prevalence of intracranial aneurysms (IAs) in the general population is estimated to be 3.6–6.0% (1). However, the incidence of SAH from the rupture of IAs is much lower at 5–20 per 100,000 (2). The mortality rate following aneurysmal SAH approaches 25–50%, with most deaths resulting from the initial hemorrhage or its immediate complications (3). Therefore, prophylactic treatment of unruptured intracranial aneurysms (UIA) is considered for selected patients considered to be at high risk for aneurysm rupture. It is important that these prophylactic treatment modalities are safe and cost-effective.

In this context, hospital readmission rates can serve as one surrogate for the safety and cost-effectiveness of preventive treatment options for UIAs. Treatment options for UIAs can be broadly categorized as endovascular (coil embolization, stent-assisted coiling, balloon-assisted coiling, flow diversion, and liquid embolics) and surgical (clipping, vessel sacrifice with or without bypass, wrapping and taping) options. Herein, we have analyzed the 30-day readmission rates and the predictors for readmission following UIA treatment with either modality.

## Materials and Methods

This retrospective study analyzed data from the National Readmission Database (NRD) derived from the Healthcare Cost and Utilization Project (HCUP) for the year 2014- this year was chosen with the consideration that the NRD database starts from 2013, and 2015 onward it contains ICD-10 codes, which we thought could have more coding variations given its more recent implementation. Ethical review and approval was not required for the study on human participants in accordance with the local legislation and institutional requirements. Written informed consent from the patients/participants was not required to participate in this study in accordance with the national legislation and the institutional requirements. The index admissions were identified by using International Classification of Diseases, Ninth Revision, and Clinical Modification (ICD-9 CM) code for unruptured aneurysm 437.3; treated with coiling 39.75, 39.76; stenting 00.65, 39.90; or surgical clipping 39.51. The treatments were subdivided into endovascular (consisting of coil embolization, stent-assisted coil embolization, and stent-placement only sub-groups) and open surgical groups.

We used the “NRD visit link (NVL)” to identify index visits and to track readmissions following the index admissions. For our purposes, “readmission” was defined as admission to particular state hospitals for any cause within 30 days of discharge following the index hospitalization. We used the sampling weights provided in the NRD database to generate the national readmission rate. Sampling discharge weights for national appraisals are produced utilizing the objective collection of community hospitals (excluding rehabilitation and long-term acute care hospitals) in the United States.

The study cohort consisted of patients admitted between January 1, 2014, and November 30, 2014, with a primary discharge diagnosis of a treated unruptured aneurysm. The patients admitted during the month of December were excluded as 30-day follow-up data to identify readmissions for these index admissions were not available in the 2014 NRD dataset. Additionally, cases with a missing or zero length of stay (LOS), and age <18 years were excluded. NRD is de-identified publicly available data exempt from institutional review boards. All the authors analyzing the database signed the HCUP data use agreement.

The following data were collected from the NRD (4): patient demographics; hospital characteristics such as bed size and teaching status; socioeconomic factors including median household income category for patient's zip code and primary payer; comorbidities, LOS, admission day, and discharge disposition. The co-morbidities were identified by ICD 9 diagnostic codes listed in secondary diagnoses fields during the index admission. The modified Charlson-Deyo Comorbidity Index (CCI) is an established measure to quantify the burden of comorbid conditions; the scores range from 0 to 33 with a higher score indicating a greater burden of concomitant diseases (5).

The primary outcome variable chosen for the study was comparison of the 30-day readmission rate (DRR) in open surgical vs. endovascularly treated groups. The secondary outcomes included predictors of readmissions, and causes of 30-DRR in these two groups. We used SAS 9.4 (SAS Institute Inc, Cary, North Carolina) for data analysis. Categorical variables and continuous variables were assessed by the Rao Scott Chi-square test and the Student's *t*-test, respectively. The predictors of readmissions were identified using multivariable logistic regression after adjusting for the stratified cluster design of NRD and co-variables including patient demographics, hospital-specific factors, socioeconomic variables, discharge disposition, and the most prevalent comorbidities to identify factors associated with readmission. The power of the model and association of predicted probabilities are determined by “concordance” statistic C which is significantly higher (>0.7) for multivariate analysis. The variables with missing or invalid values were excluded from the final analysis.

## Results

The baseline characteristics of the two comparison groups with respect to patient demograph1ics, medical comorbidities, median household income, insurance, hospital type and discharge disposition are depicted in Table 1.

**Table 1:** Baseline characteristics of study patients with UIAs who underwent surgical or endovascular intervention.

**Baseline Characteristics**	**Surgical group**			**Endovascular group**		
	**Index admits *N* = 3,533**	**Readmit N = 296 (8.37% of index admissions)**	***P*-Value**	**Index admits *N* = 3,982**	**Readmit *N* = 194 (4.87% of index admissions)**	***P*-Value**
Age (mean)	55.65 ± 0.30	57.85 ± 1.01	0.054	59.44 ± 0.38	59.00 ± 2.062	0.56
Female (%)	2,559 (72.45)	197 (66.5)	0.134	3,093 (77.67)	134 (69.0)	0.11
Charlson index (mean)	1.71 ± 0.031	1.71 ± 0.11	0.5	1.65 ± 0.029	2.04 ± 0.18	0.002
Hospital LOS (Days) (mean)	6.44 ± 0.30	7.72 ± 0.50	0.81	2.59 ± 0.14	5.91 ± 1.43	0.006
Hospital charge ($) (mean)	336,27 ± 1,387.43	382,15 ± 2,072.21	0.66	300,70 ± 1,004.10	449,32 ± 4,173.62	0.0005
**Comorbidities**						
Hypertension	2,241 (63.43)	226 (76.35)	0.002	2,443 (61.34)	121 (62.63)	0.81
Anticoagulant use	71 (2.01)	7 (2.25)	0.89	144 (3.62)	18 (9.42)	0.005
Antiplatelet use	344 (9.75)	36 (12.16)	0.29	1,156 (29.03)	70 (36.19)	0.22
Smoking	1,080 (30.57)	70 (23.75)	0.077	924 (23.19)	52 (26.72)	0.46
Alcohol	113 (3.20)	4 (1.5)	0.17	103 (2.59)	5 (2.56)	0.99
Coagulopathy	19 (0.55)	2 (0.68)	0.81	11 (0.27)	0	–
Hyperlipidemia	1,145 (32.41)	112 (37.94)	0.13	1,308 (32.86)	75 (38.45)	0.32
Diabetes	472 (13.36)	50 (16.89)	0.26	542 (13.62)	40 (20.67)	0.08
Atrial fibrillation	133 (3.77)	16 (5.34)	0.44	157 (3.95)	15 (7.63)	0.035
Ischemic stroke	201 (5.68)	18 (6.08)	0.88	131 (3.30)	15 (7.89)	0.027
Primary payer			0.01			0.41
Medicare	1,154 (32.88)	131 (45)		1,722 (43.28)	97 (50.30)	
Medicaid	555 (15.80)	52 (17.86)		528 (13.27)	30 (15.38)	
Private including HMO^‡^	1,598 (45.52)	98 (33.61)		1,517 (38.13)	58 (30.14)	
Self-pay/no charge/other	204 (5.80)	10 (3.53)		211 (5.31)	8 (4.18)	
Disposition			–			
Home	2,584 (73.17)	178 (60.20)		3,633 (91.28)	140 (72.07)	–
Home health care	500 (14.15)	51 (17.23)		148 (3.71)	11 (5.68)	
Facility	443 (12.54)	67 (22.57)		197 (4.94)	43 (22.25)	
Against medical advice	5 (0.15)	0		3 (0.08)	0	

*The numbers in parentheses indicate percentages of the respective subgroup*.

*P-values represent differences between the variables*.

*Represents a quartile classification of the estimated median household income of residents in the patient's ZIP Code, derived from ZIP Code-demographic data obtained from Claritas. The quartiles ranges are indicating the poorest to wealthiest populations. Because these estimates are updated annually, the value ranges vary by year. Derived from https://www.hcup-us.ahrq.gov/db/vars/zipinc_qrtl/nisnote.jsp*.

‡ *HMO, Health Maintenance Organization*.

*The bed size cut-off points divided into small, medium, and large have been done so that approximately one-third of the hospitals in a given region, location, and teaching status combination would fall within each bed size category. Derived from https://www.hcup-us.ahrq.gov/db/vars/hosp_bedsize/nisnote.jsp*.

The 30-day readmission rate for the surgical group was 8.37% (296 of 3,533) compared to 4.87% (194 of 3,982) for the endovascular group. The index hospitalization duration was longer in the surgical group when compared to the endovascular group (6.44 ± 0.30 days vs. 2.59 ± 0.14). A larger proportion of the patients readmitted following surgical treatment were hypertensive (76.35, vs. 63.43% of index surgical admissions), but the prevalence of other medical comorbidities was comparable in the two treatment groups. Over 90% of the endovascularly treated patients were discharged home following index admission, vs. just under three-fourths in the surgical group (Chi-square statistic 429.0,536, *p* < 0.00001).

Table 2 shows logistic regression models for outcomes of readmission in separate surgical and endovascular populations.

**Table 2:** Characteristics of readmitted patients.

	**Surgical**			**Endovascular**		
**Variables**	**Adjusted odds ratios**	**UL**	**LL**	**Adjusted Odds ratios**	**UL**	**LL**
Increased age	1.002	0.982	1.022	0.982	0.941	1.024
Female vs. male	0.759	0.491	1.174	0.656	0.336	1.281
Increased charlson index	0.891	0.701	1.132	1.231	1.016	1.49
**Median household income category for patient's zip code**						
0 to 25th percentile	Referent			Referent		
26th to 50th percentile	0.937	0.572	1.537	0.714	0.255	1.998
51st to 75th percentile	0.52	0.299	0.905	0.568	0.251	1.286
76th to 100th percentile	0.439	0.168	1.147	0.498	0.142	1.743
**Hospital bed size**						
Small	Referent			Referent		
Medium	1.657	0.968	2.838	1.533	0.561	4.189
Large	1.618	1.08	2.424	1.368	0.53	3.533
**Associated conditions**						
Hypertension	1.828	1.127	2.967	0.815	0.47	1.414
Hyperlipidemia	1.052	0.739	1.497	1.209	0.649	2.254
Diabetes mellitus	1.23	0.668	2.263	1.221	0.618	2.41
Ischemic stroke	0.991	0.386	2.54	2.412	0.849	6.856
Anticoagulant use	0.723	0.125	4.171	2.38	0.961	5.891
Antiplatelet use	1.301	0.747	2.267	1.384	0.788	2.43
Smoking	0.721	0.455	1.143	1.218	0.659	2.252
Alcohol abuse	0.482	0.143	1.629	0.691	0.172	2.769

*UL, upper limit; LL, lower limit*.

**Figure 1:**
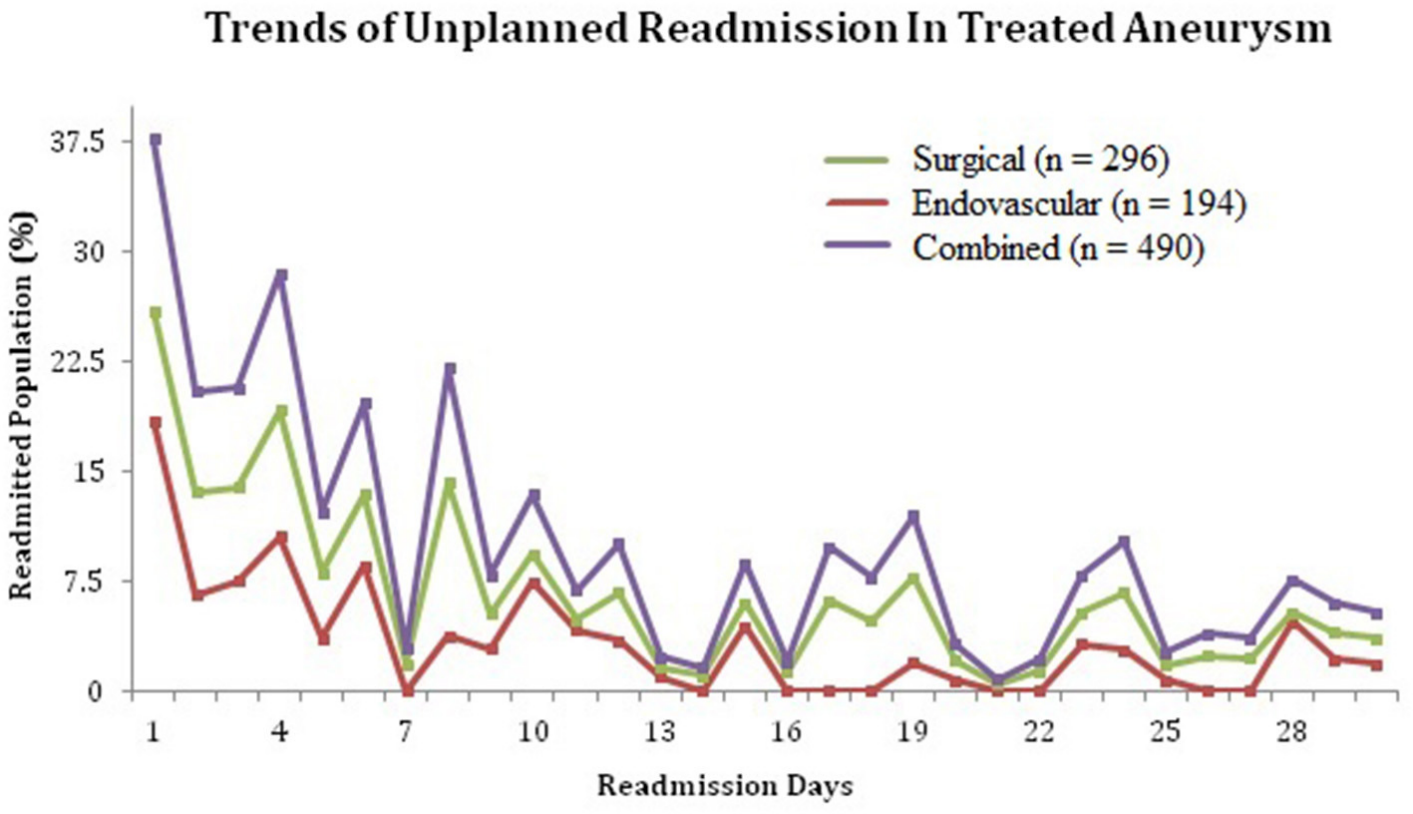
Trends of unplanned readmissions in patients treated for unruptured intracranial aneurysms.

Figure 1 shows the timing of readmission following discharge from index hospitalization for both surgical and endovascular groups.

Patients readmitted following surgical treatment had a significantly higher prevalence of hypertension [Odds Ratio 1.828 (95% CI 1.127–2.967, *p* = 0.149)] although this was not the case for other medical comorbidities. The CCI, a predictor of 10-year survival in patients with multiple medical comorbidities, was significantly higher in patients readmitted following endovascular therapy [Odds Ratio 1.231 (95% CI 1.016–1.49, *p* = 0.0338)] vs. those who were not.

Table 3 shows the combined Odds Ratios for readmission based on various patient characteristics while Tables 4A,B show the common readmission diagnoses in the two groups. As indicated in Table 3, the Odds Ratio for readmission following surgical treatment of UIAs was 1.89 times that of endovascular treatment. For both Surgical and Endovascular groups, ischemic and/or hemorrhagic complications of UIA treatment were the most common causes of readmission.

**Table 3:** All patients (surgical and endovascular) readmission model and potential contributory factors.

**Variables**	**Adjusted Odds Ratio**	**UL**	**LL**	***P*-value**
Open surgical	1.894	1.412	2.542	<0.0001
Age	0.992	0.971	1.015	0.4995
Female vs. Male	0.722	0.505	1.033	0.0742
Charlson Index	1.029	0.889	1.191	0.6979
**Median household income category for patient's zip code**				
0 to 25th percentile	Referent			
26th to 50th percentile	0.901	0.539	1.507	0.6899
51st to 75th percentile	0.568	0.353	0.911	0.0193
76th to 100th percentile	0.478	0.229	0.999	0.0498
**Hospital bed size**				
Small	Referent			
Medium	1.488	0.902	2.455	0.1188
Large	1.458	0.924	2.3	0.1044
**Associated conditions**				
Hypertension	1.303	0.955	1.778	0.0947
Hyperlipidemia	1.115	0.829	1.5	0.4698
Diabetes mellitus	1.235	0.766	1.991	0.385
Ischemic stroke	1.446	0.769	2.719	0.2515
Anticoagulant use	1.548	0.55	4.362	0.4063
Antiplatelet use	1.284	0.877	1.882	0.1981
Smoking	0.885	0.594	1.317	0.545
Alcohol abuse	0.568	0.223	1.447	0.2346

*UL, upper limit; LL, lower limit*.

**Table 4A:** Top causes of readmission in open surgical population (Weighted *N* = 296).

**Causes**	**%**
Intracranial hemorrhage	13.00
Other complications of surgical and medical procedures^*^	10.41
Postoperative infection	5.53
Unspecified septicemia	4.77
Unspecified cerebrovascular disease^®^	4.66

* *Surgical complications including malfunction, infection and inflammation of device; Medical complications including cardiac, respiratory, gastrointestinal, urinary, hemorrhage or hematoma complicating a procedure*.

® *Cerebral atherosclerosis, cerebral arteritis, moyamoya disease, non-pyogenic thrombosis of intracranial venous sinus, transient global amnesia*.

**Table 4B:** Top causes of readmission in endovascular population (Weighted *N* = 194).

**Causes**	**%**
Unspecified cerebrovascular disease	11.65
Occlusion of cerebral arteries	10.87
Other complications of surgical and medical procedures^*^	9.03
Unspecified headache	6.00
Intracranial hemorrhage	5.56

* *Surgical complications including malfunction, infection and inflammation of device; Medical complications including cardiac, respiratory, gastrointestinal, urinary, hemorrhage or hematoma complicating a procedure*.

## Discussion

Treatment paradigms for UIAs have shifted over the past two decades with a majority of patients now being treated with endovascular techniques. Currently, multiple patient and aneurysm characteristics are taken into consideration, first when choosing patients with UIAs for treatment, and subsequently for determining the best treatment approach, whether endovascular or open surgical. For the first step, various scoring systems including the PHASES score (6), have been employed to identify at-risk UIAs. However, for the next step, there is no widely adopted process for patient selection for a specific treatment.

A standardized process for determining the modality of treatment for UIAs could be very useful since both clipping and coiling can be associated with serious periprocedural complications, including arterial dissection, infarction, intracranial hemorrhage and seizures. Comprehensive knowledge of the outcomes with each type of procedure will be helpful in the development of such standardized criteria for patient selection. The outcomes of UIA clipping and coiling have been studied before with respect to aneurysm location (7), periprocedural complications (8–10), short- and long-term morbidity and mortality (11–13), expenditure (14), patient age (15), and aneurysm recurrence (16). In our study, we have focused on the readmission rates following UIA treatments with endovascular or open surgical techniques. These readmission rates can serve as a metric for the relative safety, cost and efficacy of these procedures.

Our study shows that the 30-day readmission rate following treatment of UIAs is higher with open surgical treatment than with endovascular treatment. It also shows that there is an increase in the likelihood of readmission for patients undergoing surgical treatment when there is an accompanying history of hypertension. Additionally, our study shows that the patients in the surgical group had a longer average duration of index hospitalization, and a lower likelihood for discharge to home, compared to the endovascular group. In part, at least, these findings may reflect the higher post-procedure complication rates associated with surgical clipping, which have been reported previously. We also found that there is an increased incidence of readmission due to intracranial hemorrhage after surgical treatment of UIAs. This finding could reflect the observation of development of chronic extra-axial hematomas/hygromas following clipping for UIAs, with higher rates in elderly patients (17–19). While intracranial hemorrhage was one of the top causes for readmission in both groups, certain other conditions were more specific to the type of intervention, as detailed in Tables 4A,B. Infections were a major reason for readmission in the open surgical group, while ischemic stroke seemed to be responsible for a sizeable number of readmissions in the endovascular group. These findings may reflect the complications specific to either procedure and post-procedure duration of hospitalization among other factors.

With regards to cost-effectiveness, the increased readmission rates, as well as the higher rates of initial discharge to nursing facilities shown here, are likely responsible for the increased costs associated with clipping in the initial 30-day postoperative period. Similar findings have recently been reported in a large meta-analysis comparing cost-effectiveness of the two treatment modalities (20). However, this finding of increased initial costs with surgical clipping must be interpreted with caution, given the much lower rates for aneurysm recurrences and retreatments associated with surgical clipping for UIAs, when compared to endovascular treatments. On the lines of another recently published study (21), we found lower initial complication rates with endovascular treatments, but this may also be offset by the additional morbidity and mortality risks from re-treatment procedures. In this context, the impact of potentially longer-lasting endovascular treatments such as flow diversion on long term cost reduction also needs to be studied.

Our findings must be viewed in the context of the limitations associated with the methodology of this study. The NRD does not track out of state readmissions. Similarly, NRD does not provide specific information regarding additional factors that can potentially affect the readmission rates such as aneurysm characteristics and procedural details. Additionally, the ICD-9-CM codes used in our study may have been affected by coding practices and errors. The retrospective design, implemented here using a large database not designed explicitly for this study, can only show associations. The cause of readmission is not always attributed to the procedure which is difficult to confirm in the NRD database. Different proportions of patients with UIAs with index surgical or endovascular treatment subsequently require repeat hospitalization for intervention, and this treatment complication could not be assessed using the NRD. Finally, the results could be subject to random variations as only the data from the year 2014 were included. Despite these limitations, our study included a large number of patients from a national database and is broadly representative of national estimates and predictors of 30-RR in treated UIA population.

## Summary

In the studied population, patients undergoing endovascular treatment for UIA were less likely to be readmitted within the first 30 days following treatment, and were more likely to be discharged home compared to patients undergoing open surgery. It also shows that a history of hypertension in the open surgical group appears to increase the likelihood of readmission for patients. These findings, however do not demonstrate the long term superiority of one specific treatment modality over the other. Optimal perioperative management of medical and surgical complications and careful patient selection for either intervention may help prevent avoidable readmissions following UIA treatment.

## Data Availability Statement

The raw data supporting the conclusions of this article will be made available by the authors, without undue reservation.

## Ethics Statement

Ethical review and approval was not required for the study on human participants in accordance with the local legislation and institutional requirements. Written informed consent from the patients/participants to participate in this study was not required in accordance with the national legislation and the institutional requirements.

## Author Contributions

TM, RT, and BJ were involved in the conceptualization of the study. ND, SM, AK, and AG participated in manuscript writing. TM, SM, AK, and AG were involved in collection of data. TM and SP performed the statistical analysis. TM, ND, RT, and BJ reviewed and critiqued the manuscript before presenting it in its final form. All authors contributed to the article and approved the submitted version.

## Conflict of Interest

The authors declare that the research was conducted in the absence of any commercial or financial relationships that could be construed as a potential conflict of interest.
